# Supplementary material for: ECMO with vasopressor use during early endotoxic shock: Can it improve circulatory support and regional microcirculatory blood flow?
Source: PLoS One. 2019 Oct 10;14(10):e0223604. doi: 10.1371/journal.pone.0223604 (PMC6786553; doi:10.1371/journal.pone.0223604)
Supplement: Attachment — Submitted filename: PLoS One Response to Reviewers.pdf [file pone.0223604.s001.pdf]

REPLY TO
ATTENTION OF

DEPARTMENT OF THE ARMY
BROOKE ARMY MEDICAL CENTER
3551 ROGER BROOKE DR
JBSA FORT SAM HOUSTON, TEXAS 78234-6200

29 AUG 2019

Michael Bader
Academic Editor
PLoS One Journal
1160 Battery St
Koshland Bldg East, Suite 225
San Francisco, CA 94111

Re: Manuscript: ECMO with vasopressor use during early endotoxic shock: Can it improve circulatory support and regional microcirculatory blood flow?

Dear Dr. Bader:

Thank you for the opportunity to respond to the reviewers' comments to our manuscript. Enclosed are our point-by-point responses and changes made to the manuscript.

1. Academic Editor comments.

A. Ensuring manuscript meets PLoS One's style requirements for file naming. Changes have been made to Level 1 and Level 2 Headings, Figures, Tables, and reference citations as well to the title/author/affiliation section. Your recommendations regarding where to include funding and governmental disclosure statements are appreciated.

B. Additional details in Methods section regarding animal care.

1. Method of euthanasia. Beuthanasia-D was used for euthanasia and now included in the methods section of the manuscript.

2. The source of the pigs (experimental and donor). Oshiro Farms (Waianae, HI) provided both experimental and donor pigs for this protocol. They are referenced in the methods section of the manuscript.

3. Clarification re: anesthesia. The pigs were anesthesized the entire duration of the experiment and never regained consciousness. This statement is also included in the manuscript's method section.

2. General Comments from Reviewer #1.

A. "A group with shock and vasopressors without ECMO would have been interesting" Thank you for this interesting observation. We agree that a 5th group would have allowed for more comparison and contrast of our data. However, our protocol was not designed to include an additional arm to this study as we felt that data are already known about the use of vasopressor in shock states.

3. General comments from Reviewer #2.

A. Is the manuscript technically sound and do the data support the conclusions? "Partly". We appreciate Dr. Kjaergaard's extensive and very thoughtful review of our manuscript and have made many edits as a result of his comments and suggestions. These are noted further down in this response letter.

B. Statistical analysis performed appropriately and rigorously. "I don't know". The investigators on this project have extensive statistical experience along with research design. A statistical analysis paragraph is included in the body of this manuscript.

4. Specific Comments Reviewer #2.

A. Line 46 MODS abbreviation. We have deleted this abbreviation as suggested.

B. Even though local authorities approved the study, the anesthesia was not in accordance with European standard. In Europe, a protocol with Pentobarbital without analgesics cannot be accepted in surgery. Was the anesthesia as described or is anything missing? Thank you for allowing us to clarify this aspect of our protocol which has been incorporated into the manuscript in lines 83-87. Anesthesia was administered in accordance with American Veterinary Medical Association guidelines. Before induction of anesthesia, pigs were sedated with the following regimens: acepromazine 1.1 mg/kg IM plus Ketamine 22-33 mg/kg IM plus atropine 0.02-0.25 mg/kg IM or with a sedation dose of pentobarbital 30-50 mg/kg intraperitoneal. Anesthesia was maintained with pentobarbital as the anesthetic agent and pigs never regained consciousness throughout the experiment. Alternatively, our lab more recently uses an anesthesia regimen of ketamine, midazolam and sufentanil cocktail, but for the animals used in this study, pentobarbital was the primary anesthetic agent.

C. Provide additional information re: ventilator settings during the experiment, FiO2, oxygenator and ventilator flow adjustments. We appreciate the request for additional information and have included in the manuscript in lines 123-126. We aimed to avoid any confounding effects of increased FiO2 on hemodynamic parameters and thus we maintained 21% FiO2 for the time period preceding any lab or hemodynamic measurements. If at anytime a brief increase in FiO2 was needed to support the animal to prevent development of hypoxia, it was delivered only to stabilize the animal and then returned back to 21% FiO2 before any measurements were obtained. Our conventional ventilator settings were initially set to support physiological tidal volume requirements to maintain normal pCO2. Once the animal was on ECMO, we placed the animal on "rest" settings which consisted of lower respiratory rates, maintaining PEEP. Oxygenation on ECMO was set to 21% FiO2 and sweep gas rates (300-400ml/min) adjusted to maintain pCO2 between 35-45mmHg.

D. Line 107 Please explain cephalad 8-10 Fr catheter in the jugular vein. In order to achieve flow rates of 80-100 ml/kg/min on ECMO, we needed additional venous return and thus used this cephalad catheter. We have added a clarification phrase into the manuscript. (line 112)

E. Line 108 Arterial cannula placement at the left ventricle. Thank you for this excellent pick-up and comments about the adverse effects of misplaced arterial cannulas. We have corrected this error in the text. The arterial cannulae were placed at the aortic arch (line 112)

F. Line 96 Another catheter placed in the same internal carotid artery and placed in the left ventricle. As Dr. Kjaergaard has assumed correctly, we have added the size of this catheter (3.5 Fr) that was used for left ventricular pressure monitoring as well as colored microsphere injections. (line 100)

G. 450ml priming volume is very big for a 9kg pig even the circuit was primed with blood. The donor blood can interfere with pulmonary resistance. Did the control group not on ECMO receive donor blood? Our ECMO circuit configuration (oxygenator, pump, and tubing length) required over 400ml to prime. The control group (group 1) did not receive any donor blood. Thus the effect of putting the animal on ECMO includes the effects of blood priming of the circuit.

H. Why not use an infant set for ECMO or bigger pigs instead of using a 50kg pig as donor. At the time when we first started these experiments, we did not have an infant ECMO set. We did eventually get the infant sets that only required about 250 ml to prime the circuit. When we did switch to the smaller sets, we did not notice any differences in physiological responses from the adult ECMO set of filter and tubing. Thus, we do not believe there was an appreciable influence of the circuit blood volume on the pigs in this study. We chose 50kg pigs to harvest blood for use primarily for two reasons. The first reason was to use same species blood for ECMO. Secondly, while larger pigs were available for purchase, the 7-9 kg pig size was used as the results from this study could be compared against results from other studies in our lab. Using this size animal model, we found the physiological responses between the 7-9 kg range and 50 kg range pigs were similar, and so using the smaller pigs was more practical in managing the pigs.

I. If the extracorporeal system as biocoated, is it possible to run the ECMO with ACT below 200 seconds. Our protocol stipulated targeting ACT between 150-200 and it is included in the manuscript. Our team's experience working with pig blood is that it is highly coagulable and thus our protocol is designed to mitigate increased risk for clotting.

J. The first group… please indicate that pigs were prepared for ECMO with all catheters inserted but ECMO was not started. We have clarification as suggested. (line 131)

K. Line 125 Please mention how many in each group first and then [discuss] ET treatment. Thank you for this recommendation. We have made edits to this section (lines 133-135) as suggested before launching into the description re: ET treatment.

L. Why did you end at 27 pigs? How many pigs did you have from the beginning? We do not understand the concern with studying a total of 27 pigs. If the reviewer is believing the preference is to have "an equal number" in each of the groups, we respectfully counter that is not necessary nor desired when doing a truly randomized experiment while using as few animals as possible once statistical power is sufficient to discern differences between groups. The protocol was designed to achieve an "n" of 6 to 8 in each study arm based on statistical power estimates of anticipated physiological variability. It was also important that we randomized the animals to each group so as not to have a bias in the order the experiments were performed. Once statistical power was achieved with the actual data obtained, and statistical differences were demonstrated between groups, we are required to stop the study to assure minimal animal usage (a tenet of IACUC approval). In addition, the creation of the

endotoxic shock steady state is difficult to achieve, and only data from animals that achieved the same degree of steady state hypotension were used to reduce the variability of the animal model so we could be confident that differences in responses were due to treatment effects and not to endotoxic shock variation. Because we kept the criteria for endotoxic shock stringent, we were able to increase sample power and not have to perform experiments on 8 animals in every group. The data discussed in this manuscript reflect, in our opinion, accurate representatives of each study arm.

M. Line 190. Be careful not to conclude something of PAP, but only about PVR, because ECMO in itself will bypass blood from the pulmonary circulation. We concur with this comment and have removed the comment on PAP from the text (Line 201)

N. Line 219. Maybe better to change CO with systemic circulation. We made the change as suggested. (Line 229)

O. Line 255-270 is strictly not discussion but introduction. We appreciate this perspective. In fact, earlier versions of our manuscript had these comments in the introduction section. However, we felt that the overall manuscript flowed/read more effectively by moving it into the discussion section as a lead-in to discussion about our findings re: the interaction between ECMO and use of vasopressors on regional blood flow and oxygen delivery.

P. The biggest limitation is the species difference. We acknowledge this additional limitation and have included it into the limitations paragraph of this manuscript. (Lines 327-329)

5. In closing, we appreciate PLoS One's interest in our manuscript along with efforts made by the reviewers to improve the manuscript in consideration for publication. Please contact me if there are additional concerns and/or recommended revisions. Thank you for your time and attention to our manuscript, and we await your response.

Sincerely,

Thornton S. Mu, MD, FAAP
